# Deep dorsal penile vein thrombosis in a patient with COVID‐19 infection: A rare complication and the first reported case

**DOI:** 10.1002/ccr3.5117

**Published:** 2021-12-06

**Authors:** Seyed Morteza Bagheri, Zhale Tabrizi

**Affiliations:** ^1^ Department of Radiology Hasheminejad Kidney Center (HKC) Iran University of Medical Sciences Tehran Iran; ^2^ Radiology Department Iran University of Medical Science Tehran Iran

**Keywords:** COVID‐19, deep dorsal penile vein thrombosis, deep vein thrombosis, hypercoagulopathy, ultrasound

## Abstract

COVID‐19 infection may have extrapulmunary manifestations such as blood hypercoagulability that may cause thrombosis in both arterial and venous system. Deep dorsal penile vein thrombosis is very rare, and the most common reason is coagulation disorders. The common observed symptom is penile pain especially during erection and it is diagnosed by ultrasound evaluation of the vein. It is necessary to distinguish deep dorsal penile vein thrombosis from superficial dorsal penile vein thrombosis as it needs anti‐coagulant treatment. In present study, we describe a unique case of the deep dorsal penile vein thrombosis following COVID‐19 infection.

## Graphical abstract

COVID‐19 infection can cause hypercoagulopathy, so it can cause thrombosis in deep veins, mostly in lower limbs. But in this paper, we describe a patient with deep dorsal penile vein thrombosis following COVID‐19 infection. This is a very rare condition.

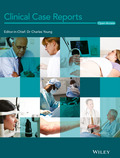

## 1 INTRODUCTION

Venous thrombosis of the penis is often related to thrombosis of the superficial veins (Mondor disease), that has been well reported in the literature in the last decades.^1^ While Thrombosis of the deep dorsal penile vein has been rarely reported.^2^ These two conditions need different treatments, for instance, mondor disease can be cured simply through or after treating by anti‐inflammatory or anti‐coagulant drugs. But thrombosis of the deep veins may cause serious complications such as ischemia and priapism, therefore it needs to be treated by anti‐coagulants. To this end, It is important to distinguish the two conditions from another.^3^, ^4^

Although COVID‐19 is mostly known for its respiratory symptoms, it can also have many extrapulmonary manifestations, such as thrombotic complications, mostly pulmonary embolism and deep venous thrombosis.^5^, ^6^

In the present study, a unique case of deep dorsal penile vein thrombosis following COVID‐19 infection is reported for the first time.

## 2 CASE PRESENTATION

The case study is devoted to investigating of penile pain in a 41‐year‐old married man. According to medical evaluation, the pain extended to the perineal and inguinal regions and it was reported to be more acute during erection. The patient was referred by urologist for sonographic evaluation of penis and testes. The pain had started 3 days before the urologist examination, following his first full erection for intercourse, after his positive COVID‐19 polymerase chain reaction (PCR) test.

The patient did not have any other urologic symptoms such as discharge, hematuria, or dysuria. He denied any trauma to the penis, previous pelvic tumor, pelvic surgery and history of recent immobilization. He did not use vasoconstrictive drugs. The patient reported positive nosopharyngeal swab test for COVID‐19 three weeks earlier. He had mild symptoms of COVID‐19 infection including muscle pain, fever, cough, and fatigue. He had received conservative treatment and had not taken any anti‐coagulants, antivirals, and corticosteroids. His medical history did not show any significant underlying disease and any risk factor for cardiovascular disease. He also did not have history of previous deep vein thrombosis. In physical examination of the penis and testes, no pathologic finding was detected such as skin tissue changes, discoloration, edema, tenderness, or palpable nodularity.

**FIGURE 1:**
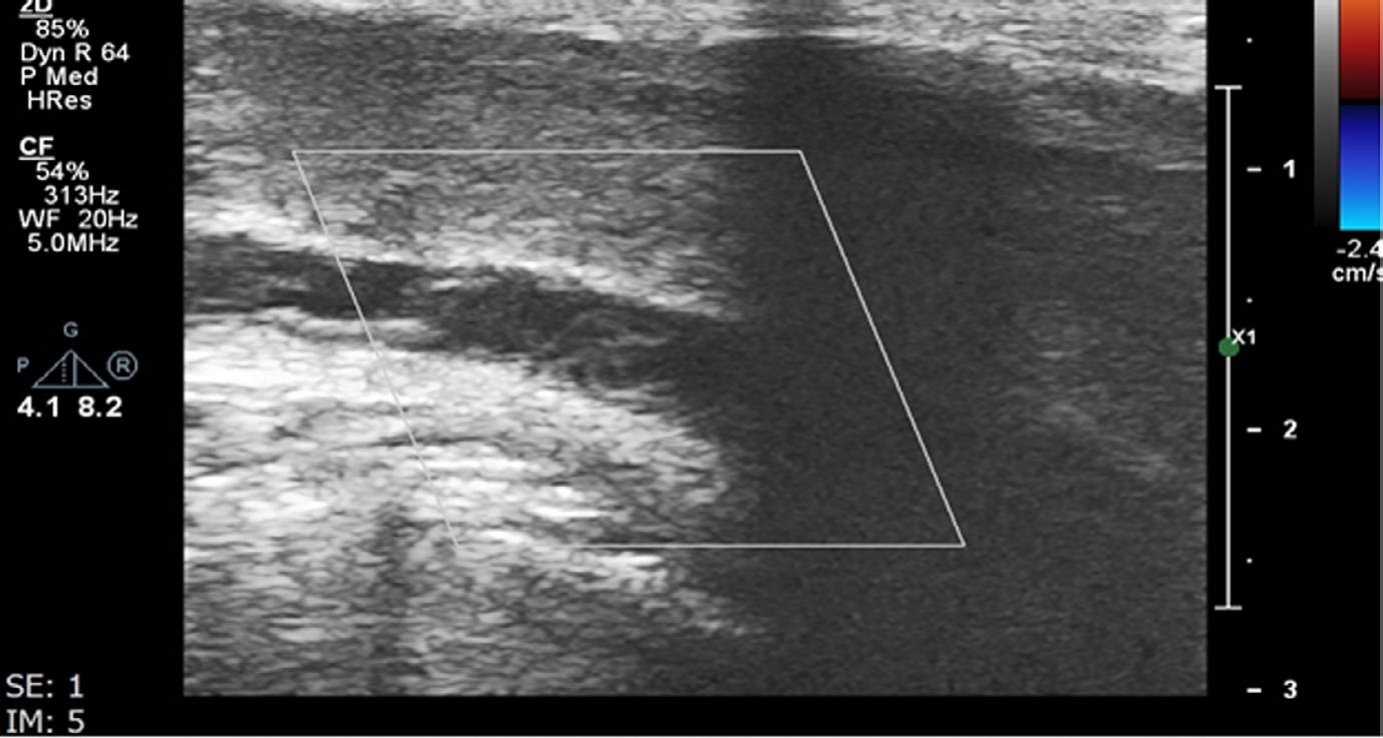
Ultrasound evaluation of the deep dorsal penile vein shows no flow in the vein. Also the vein is dilated and contains echogenic thrombosis from the middle part of the penis extending to the root of the penis at posterior of the pubic symphysis. The thrombosis is not extended to the superior of the urogenital diaphragm. Above findings are in favor of subacute thrombosis of the deep dorsal penile vein

**FIGURE 2:**
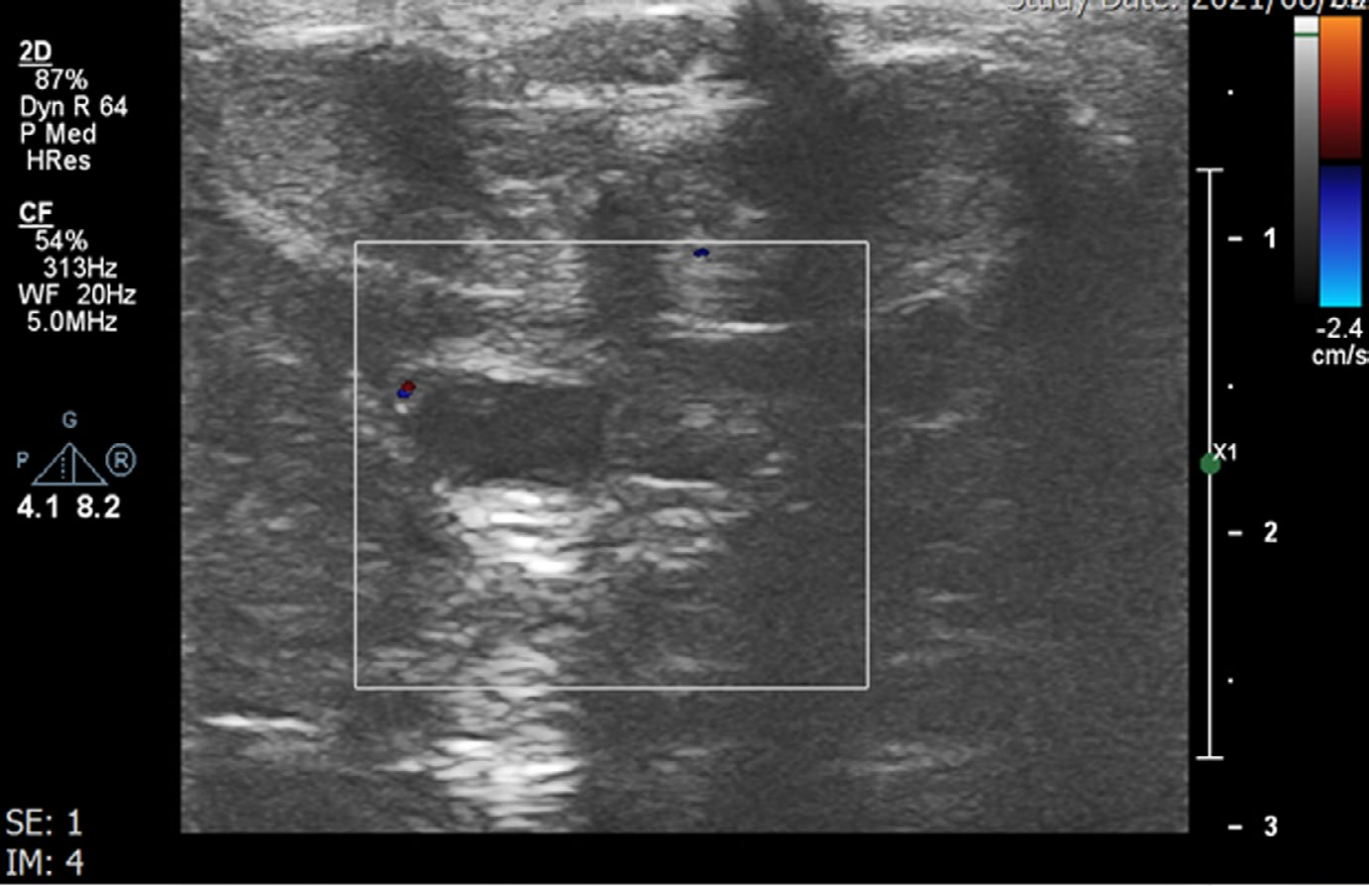
Ultrasound evaluation of deep dorsal penile vein (Arrow) shows no color in the vein. The vein contains echogenic thrombosis. White Stars: Normal right and left corpus cavernosum. Black star:Normal corpus spongiosum

**FIGURE 3:**
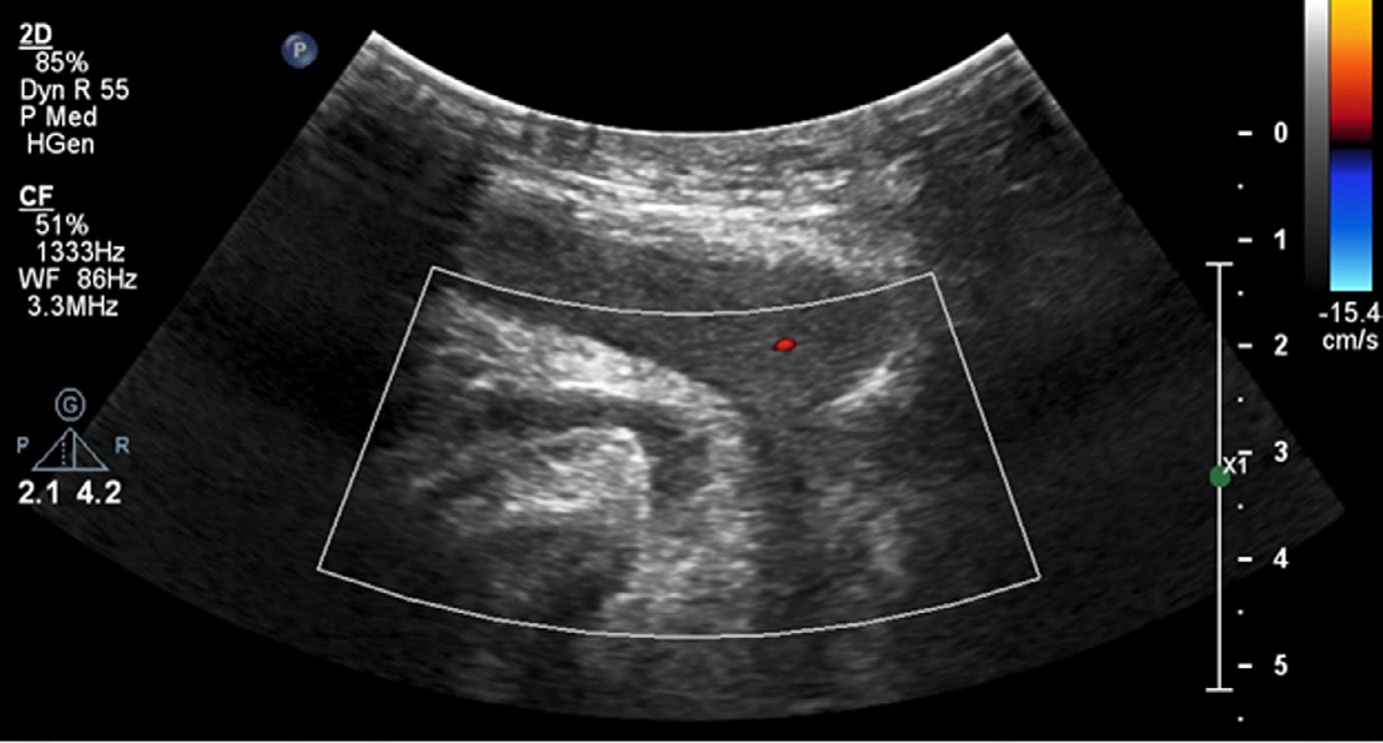
Ultrasound evaluation of the deep dorsal penile vein (Arrow) shows no flow in the vein.star: corpus spongiosum

Ultrasound evaluation showed thrombosis of deep dorsal penile vein while the superficial dorsal penile vein, iliac veins, and inferior vena cava were intact (Figures 1,2,3).

Laboratory tests revealed slightly increased D‐dimer level(may be due to inflammatory process of COCID‐19 infection), normal levels of fibrinogen, anti‐thrombin III, protein S, Protein C, anti‐cardiolipin antibodies and normal count of platelets and white blood cell counts. Also Tests were negative for anti‐phospholipid‐IgG, IgM, and lupus anti‐coagulant (Table 1).

**TABLE 1:** Lab datas

Test	Patient's result	Normal value
Lupus Anti‐coagulant	34	≤40 s
D‐Dimer	600	Up to 500 ng/ml
Fibrinogen	250	200–400 mg/dl
Anti‐thrombin III	108	80%–120%
Protein C	80	65%–145%
Protein S	72	63.5%–167.9%
Ferritin	45	32–501 ng/mL
Anti‐cardiolipin (IgM)	8.2	Negative: <12 MPL/ml Borderline: 12–18 Positive: >18
Anti‐cardiolipin (IgG)	7.4	Negative: <12 GPL/ml Borderline: 12–18 Positive: >18
Anti‐phospholipid (IgM)	2.3	Normal: <12 U/ml Borderline: 12–18 Positive: >18
Anti‐phospholipid (IgG)	3.4	Normal: <12 U/ml Borderline: 12–18 Positive: >18
Platelets count	191,000	140,000–440,000
White Blood cells count	7,100	4,400–11,300
P.T.T	33	30–40 s
P.T(Prothrombin Time)	12	13 s
I.N.R	1	0.9–1.2

**FIGURE 4:**
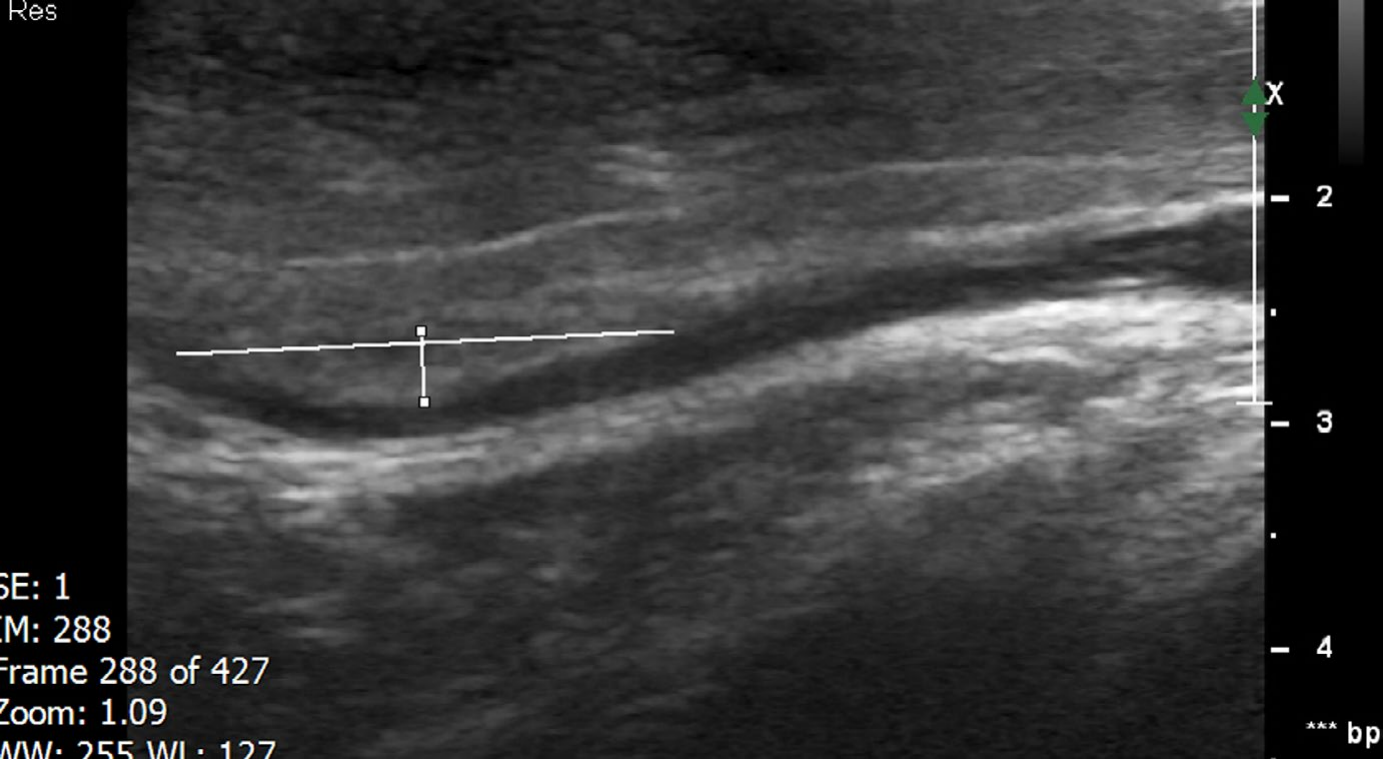
Two Months after starting the treatment. Ultrasound of the deep dorsal penile vein revealed no evidence of acute thrombosis. The vein is seen with normal diameter. At the proximal part of the vein, the dorsal wall is thickened and an old partial thrombosis measured 26 × 2.4 mm is noted. Other parts of the vein showed normal velocity with no other partial thrombosis. The vein got expanded with valsalva maneuver. Patient had a little pain with the pressure of ultrasound probe at the site of the partial thrombosis

**FIGURE 5:**
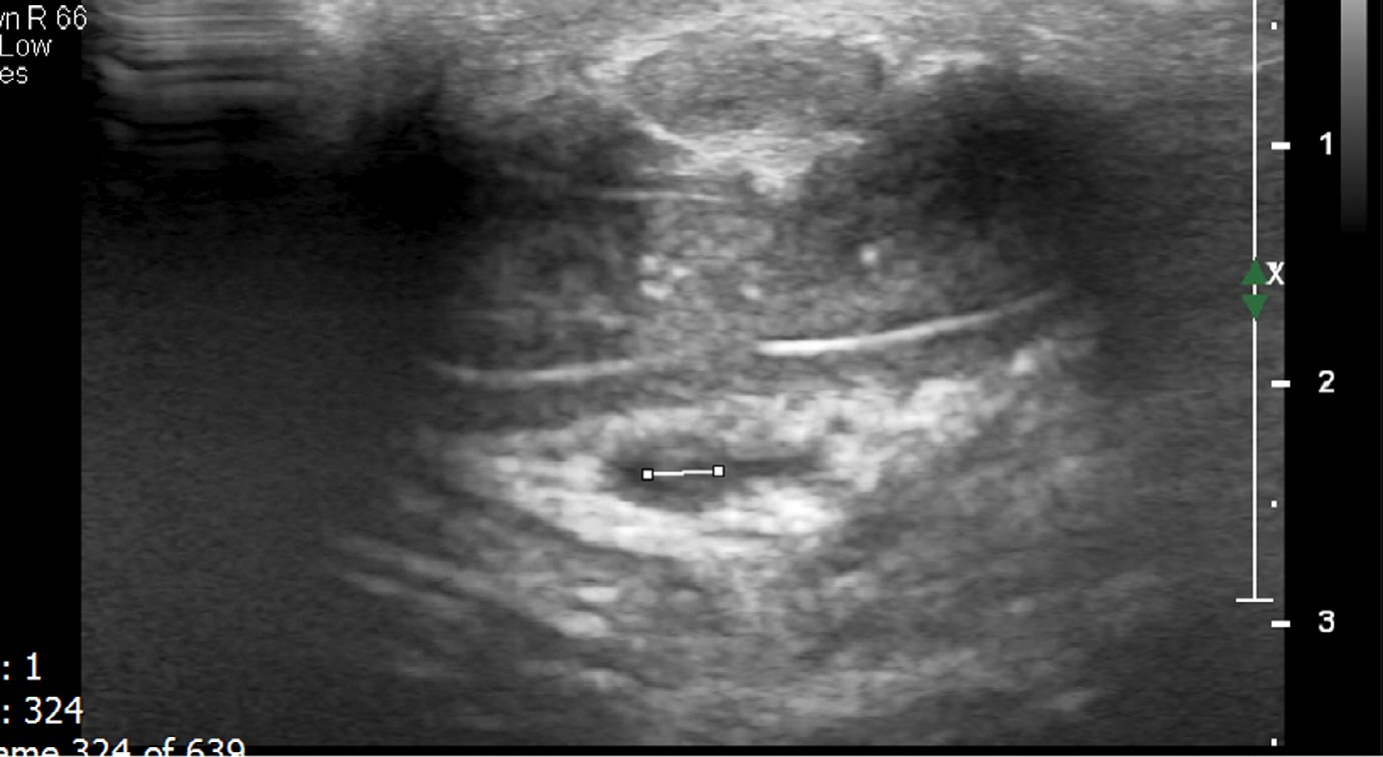
Two Months after starting the treatment. Ultrasound of the deep dorsal penile vein revealed no evidence of acute thrombosis. The vein is seen with normal diameter. At the proximal part of the vein, the dorsal wall is thickened and an old partial thrombosis measured 26 × 2.4 mm is noted. Other parts of the vein showed normal velocity with no other partial thrombosis. The vein got expanded with valsalva maneuver. Patient had a little pain with the pressure of ultrasound probe at the site of the partial thrombosis

**FIGURE 6:**
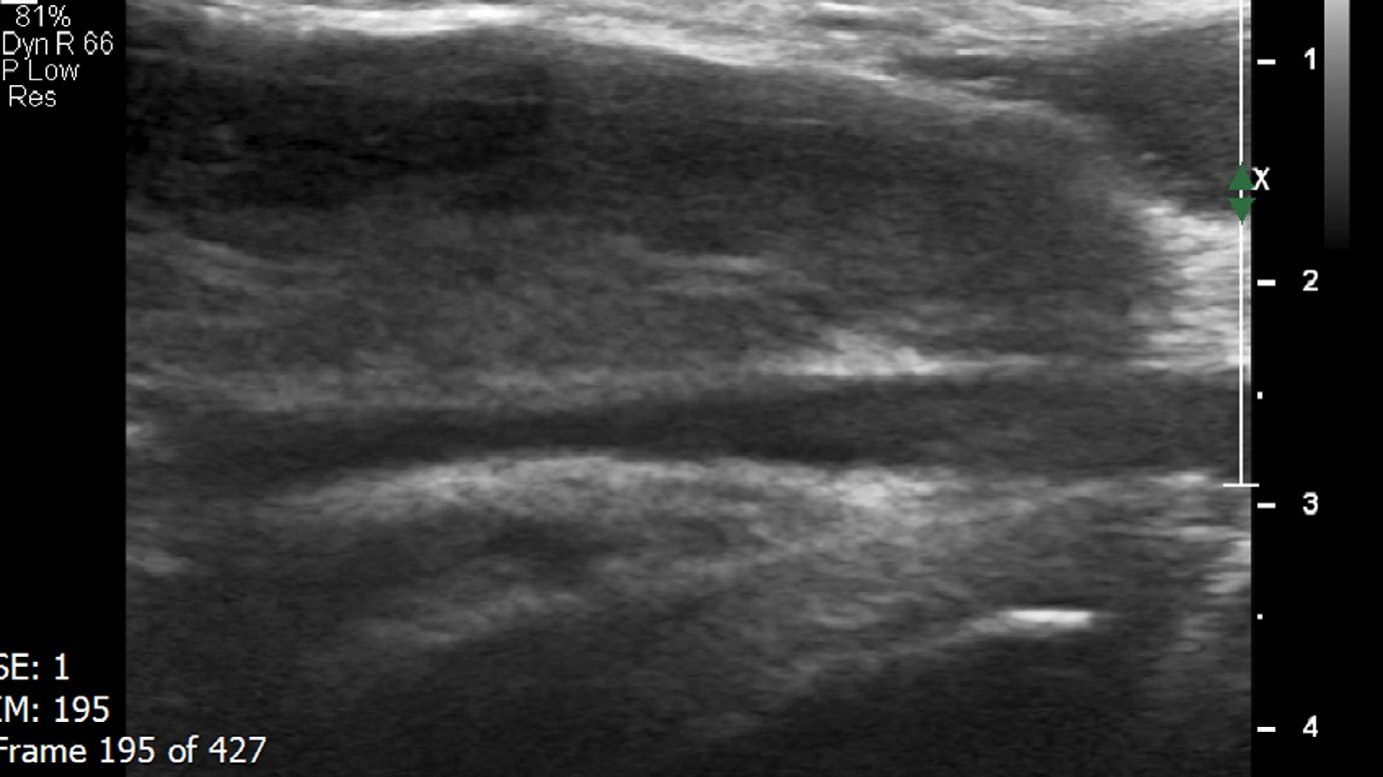
Two months after starting the treatment. There is no evidence of thrombosis at the deep dorsal penile vein except the proximal part of the vein showed in Figures 4,5. The vein is seen with normal diameter and velocity

Immediately after sonographic diagnose of deep dorsal penile vein thrombosis, the Rivaroxaban treatment was started with the dosage of 15 mg twice a day. Two months after starting the treatment, patient's symptoms were completely disappeared and he had no penile pain during erection and sexual disturbances anymore. Ultrasound evaluation revealed no evidence of acute deep dorsal penile vein thrombosis. Old partial thrombosis at the proximal part of the vein was seen. A little pain at the site of the partial thrombosis with the pressure of the ultrasound probe was noted (Figures 4,5,6).

## 3 DISSCUSION

The coronavirus is mostly known to cause pulmonary disease such as pneumonia or acute respiratory distress syndrome; however, during the course of COVID‐19 pandemic, it exhibits some extrapulmunary manifestations including hematologic, neurologic, cardiovascular, gastrointestinal and renal manifestations.^7^, ^8^, ^9^, ^10^, ^11^ This pathology may be due to extracellular proliferation of the COVID‐19 virus and the induction of immunopathological reactions, as it has been reported previously for other zoonotic coronaviruses in the literature.^12^

Coagulopathy as a part of the systemic inflammatory response syndrome is a prevalent feature of COVID‐19 infection. Roughly, 20%–50% of hospitalized patients with COVID‐19 infection have abnormal coagulation tests (elevated D‐dimer, prolonged prothrombin time, prolonged clotting time, thrombocytopenia and low fibrinogen levels) which can cause thrombosis and microvascular occlusion, due to endothelial dysfunction, cytokine storm, hypoxia, and immobilization.^13^, ^14^, ^15^, ^16^, ^17^, ^18^, ^19^ Increased D‐dimer leads to consumption of natural coagulation inhibitors that can cause vascular thrombosis.^20^

Venous drainage of the penis is performed through the following pathways: (1) The superficial dorsal venous system, which is responsible for draining the skin and soft subcutaneous tissue. The superficial dorsal venous system eventually drained in to the external pudendal vein that is anatomically linked to the long saphenous vein at the groin and (2) The deep dorsal venous system, which is responsible for draining of the glans, corpus spongiosum and the distal two thirds of corpora cavernosa. The deep dorsal venous system eventually drained in to the prostatic and perivesical venous system.^4^, ^21^

The pathogenesis of thrombophlebitis is the well‐known Virchow triad which was introduced in 1845: (1) Endothelial vascular wall damage, (2) venous stasis and (3) changes in blood components.^22^

The cause of thrombosis in the penis is mostly thrombosis of superficial venous system, which is known as Mondor disease. But deep vein thrombosis has been reported very rarely in the literature.^4^

The etiology of Mondor disease is unknown, but some etiologies listed below are reported in the previous studies: sexual activity, infection, penis injection, direct trauma, cancer, and a deficit of anti‐thrombin III, Protein C and S and previous surgical hernia repair.^3^ Patients with Mondor disease are usually asymptomatic but some of them may have penile pain, erythema of the penis or palpable superficial thrombosed vein. Generally, Mondor disease is self limiting and just need conservative treatments, and in most cases it will not have any complications.^2^ As mentioned before, deep dorsal penile vein thrombosis is very rare compared to the superficial vein thrombosis and the most common cause of spontaneous dorsal deep vein thrombosis is coagulation disorders without inflammatory processes. Since this vein is a part of deep venous system, thrombosis of deep dorsal penile vein can increase the risk of pulmonary embolism leading to significant increase in mortality and morbidity.^23^, ^24^ So investigation for other sites of thrombosis and starting appropriate treatment is necessary.^24^, ^25^

Searching the literature showed no previously published similar case of deep dorsal penile vein thrombosis fallowing COVID‐19 infection and our patient is the first reported case. Only superficial dorsal penile vein thrombosis are reported previously.^19^, ^25^ (By Ern MT et al and Levine ML et al).

The association between deep dorsal penile vein thrombosis and COVID‐19 infection has not been well clarified in the literature.

## 4 CONCLUSION

COVID‐19 infection can cause coagulopathy due to cytokine release and immunopathological reactions. In some hospitalized patient it can cause pulmonary embolism or deep vein thrombosis, but in non‐hospitalized patients can cause small deep vein thrombosis. So it is important for clinicians to investigate the risk of thrombosis in COVID‐19 patients especially those with risk factors or previous deep venous thrombosis. It is necessary to do further studies and realize if it is necessary to add prophylactic anti‐coagulant to treatment regimen of COVID‐19 patients who are not hospitalized or not.

## CONFLICT OF INTEREST

The Authors declare that there are no competing interests.

## ETHICS APPROVAL

All ethics standards and consent were obtained for this manuscript.

## CONSENT

Written informed consent for publication was obtained from the patient.

## Data Availability

Data sharing not applicable—no new data generated. Data sharing is not applicable to this article as no new data were created or analyzed in this study.
